# Combined effects of physical activity and life events on depression and PTSD in Chinese students aged 16–24 years

**DOI:** 10.3389/fpubh.2024.1449391

**Published:** 2024-08-30

**Authors:** Zhifeng Wang, Fengyun Wang, Bing Jiang, Haihong Xue, Ming Zhao, Dongmei Wang

**Affiliations:** ^1^Department of Physical Education, Xi’an Polytechnic University, Xi'an, Shanxi, China; ^2^College of Sports Medicine and Rehabilitation, Shandong First Medical University and Shandong Academy of Medical Sciences, Tai’an, China

**Keywords:** physical activity, life events, Chinese students, depression, PTSD, combined effects

## Abstract

**Background:**

Life events are important risk factors for depression and post-traumatic stress disorder (PTSD). Physical activity is a beneficial behavior to physiological and psychological health. While it has not been reported at present the combined effect of physical activity and life events on individual depression and PTSD, and whether it can alleviate the psychological risks induced by life events.

**Objective:**

To comprehensively investigate the current status of life events experiences in Chinese students aged 16–24 years and analyze the combined effects of physical activity and life events on their depression and PTSD.

**Methods:**

An online cross-sectional survey was conducted on physical activity levels, life events experiences, depression and PTSD of 1,552 Chinese students aged 16–24 using short version of International Physical Activity Questionnaire (IPAQ-S), adolescent self-rating life events checklist (ASLEC), PTSD Check List-Civilian Version (PCL-C) and Patient Health Questionnaire Depression Scale. Then, logistic regression equation and stratified analysis were used to explore the combined effects of physical activity and life events on depression and PTSD.

**Results:**

Regression analysis showed that, except for female, <8 h of sleep, smoking, single parent/reorganized families and poor family economic status, experiencing medium-intensity and high-intensity life events were both risk factors for depression. Compared with those who experienced low-intensity life events, those who experienced medium- and high-intensity life events had a 27 and 131% increased risk of depression, respectively. In contrast, medium- and high-level physical activity could reduce the risk of depression by 49 and 53%, respectively. Similar results were obtained with PTSD as a dependent variable. Combined correlation analysis showed that, compared with those with high-level physical activity and low-intensity life events, those with low-level physical activity and high-intensity life events had a 209 and 121% increased risk of depression and PTSD, respectively. Stratified analysis showed that the threshold for life events induced depression and PTSD rose with the increase in the level of physical activity.

**Conclusion:**

Lack of physical activity and experience of high-intensity life events are independent risk factors for depression and PTSD, and strengthening physical activity can compensate for the harm of depression and PTSD caused by life events to some extent.

## 1 Introduction

Life events refer to the sum of various stimuli that people face in their life, work, study and social support systems. In recent years, whether young students in school or adults in the workplace are inevitably faced with various forms of life events from natural and social environments with the accelerated changes of human natural and social environments, as well as the increasingly fierce social competition. Life events, as a stressful stimulus, are frequent and long-lasting, although they are not as strong as major geological disasters (earthquakes), climate disasters (hurricanes, rainstorms), violent conflicts and public health (COVID-19). Under such frequent and long-lasting life events, people will inevitably have a variety of bad psychology such as stress, frustration and anonymous. Therefore, the impact of life events on individual psychological health cannot be ignored. A cross-sectional survey showed that life events and parenting styles may have impacts on psychological health, and punitive education and overprotection of fathers may be risk factors for children’s psychological health (1). Stress-related life events are closely related to the occurrence of depression in adolescents (2). More stressful types of life events, negative relationship changes, death or illness of friends/family members, work-related difficulties and feeling insecure in the community were independently related to at least one of the psychological health outcomes assessed such as anxiety, depression or post-traumatic stress disorder (PTSD) (3). A 3-year longitudinal tracking study also showed a strong correlation between negative events and anxiety disorders, especially injury, illness or death of family members or close friends (4). Relatively consistent results were also reported in many previous studies (5–8). Stressful life events related to COVID-19 had important predictive effects on depression and anxiety in college students (9). Life events play a central role in the development, impact course and outcome of psychological disorders (10). Social life events are closely related to individual psychological health. To some extent, individuals with poor or moderate psychological health are less likely to experience positive life events such as improving their educational level, getting married, working and becoming parents. On the contrary, they are more likely to experience negative life events such as becoming unemployed, divorced or widowed (11).

In addition to the common psychological symptoms of anxiety, depression, and psychological stress, scholars have also studied the relationship between life events and PTSD, a severe psychological disorder (12). It was found that most patients with PTSD had experienced or suffered continuous or repeated traumas, such as child abuse, family or community violence, suggesting a close relationship between PTSD and traumatic life events (13). Women with higher postpartum PTSD (PP-PTSD) symptoms had previously experienced physical and sexual assault and child abuse (14). In a study of adolescents who had experienced earthquakes, stressful life events were found to be a better predictor of PTSD in adolescents than earthquake exposure (2). Individuals who had experienced early negative life events generally had higher PTSD detection rates, as well as higher scores for anxiety and depression (15). Stress-related life events (SLEs) were associated with PTSD and other psychological symptoms (16–20). It can be seen that life events are closely related to individual psychological health, especially negative life events are important factors leading to various psychological disorders such as anxiety, depression and PTSD. Unfortunately, life events are almost inevitable in human life, and they are characterized by frequency, persistence and unpredictability. Therefore, it is necessary to explore simple, effective and easy-to-operate methods to reduce or alleviate the adverse effects of life events on psychological health.

Physical activity refers to any physical movement caused by skeletal muscle contraction that results in energy consumption. It has been proven that physical activity has a unique role in maintaining physical and psychological health, improving psychological disorders, relieving psychological stress and assisting in the treatment of psychological disorders such as anxiety and depression (21). Physical activity could not only directly fight depression (22, 23) and anxiety (24, 25), and relieve PTSD (26), but also indirectly reduce the risk of psychological disorders by enhancing physical fitness and function, such as enhancing human cardiorespiratory fitness (27). Physical activity also showed good application value in individual psychological interventions after traumatic events caused by various major natural disasters, accidents, and public health and social security events (28). Compared with psychotherapy and pharmacotherapy, physical activity has significant advantages such as low “stigmatization,” few side effects and strong popularity (29). All of the above studies showed that physical activity can be an effective way to alleviate or combat various mental health problems caused by life events. At present, research in this field mainly focuses on the relationship between physical activity and individual mental health under extreme traumatic events. For example, studies of individuals who had experienced earthquakes (30), Fukushima nuclear accident (31), COVID-19 (32), floods and hurricanes (33), terrorist attacks (34) and war (35) found that physical activity had a positive effect on their mental health. However, all of the above traumatic events are relatively rare life events that most people may not experience once in a lifetime. On the contrary, it may be of wider application value to explore the regulating effect of physical activity on mental health problems induced by daily life events in the general population. However, few studies have been reported in this field.

This study focused on the students aged 16–24 years in school to explore the correlation between life events and physical activity and depression and PTSD, and to analyze their combined effects on depression and PTSD.

## 2 Methods

### 2.1 Study objects

A total of 1,696 questionnaires were obtained by random sampling from seven universities and some middle schools in Shaanxi, Henan, Hunan, Xinjiang and Jiangsu and other provinces in China in the form of mobile phone questionnaire star. Excluding invalid questionnaires, a total of 1,552 valid questionnaires were collected, with an effective rate of 91.51%.

According to Chinese law, the survey of middle school students obtained the consent of students, their parents and school administrators. The survey of college students obtained the consent of school administration and themselves. After clicking the link, the respondents read the electronic informed consent form first. If they agree to participate, click “agree” to fill in the electronic questionnaire; if they disagree, click “disagree” and exit automatically. All respondents have the right to withdraw from the study at any time without penalty. This study was approved by the Ethics Committee of Xi’an Polytechnic University with approval number 2022TY0029.

### 2.2 Measurement

#### 2.2.1 Short version of international physical activity questionnaire

The short version of international physical activity questionnaire was used to evaluate physical activity level of the respondents. IPAQ-S is suitable for people aged 15 to 69 years and is mainly used for demographic monitoring of physical activity levels. It has been verified in Chinese, showing good reliability among Chinese adolescent students (36). According to the literature (36–38), the respondents were required to self-report the frequency and daily duration of low-intensity (3.3 MET/min), medium-intensity (4.0 MET/min) and high-intensity (8.0 MET/min) physical activities in the past half a month. The metabolic equivalent (MET) for each intensity of physical activity was calculated separately (MET assignment × weekly frequency × daily duration), and then the MET for each intensity of physical activity was added together to obtain the overall level of physical activity. According to the classification criteria for physical activity level in the scale, the physical activity level of the respondents was divided into three categories: low (<600 MET-min/wk), medium (600–1,500 MET-min/wk) and high (>1,500 MET-min/wk).

#### 2.2.2 Adolescent self-rating life events checklist

ASLEC was used to assess the frequency and intensity of life events experienced by the respondents. The scale includes 27 items covering interpersonal conflict, family conflict, academic stress, punishment by guardians or teachers, health problems, humiliation, loss of money, and death or illness of family members. These items are grouped into six negative life events: relationships, academic stress, punishment, loss, health adaptation and others. The 5-point Likert scoring method was used for each item. The respondents were asked to answer whether these life events had occurred in the past 12 months. If the life event did not occur, the score was 0; if the life event occurred, its impact needed to be assessed, from 1 (not at all) to 5 (very much). The total score of the scale ranges from 0 to 135, and the higher the score, the more severe the impact of life events on individuals. It has been demonstrated that the scale has good reliability and validity in the Chinese population (39). In the present study, the Cronbach’s α was 0.90.

According to the quartile distribution of the final ASLEC survey results, the life events experienced by the respondents were divided into four categories: very low-intensity life-events: 0–45; low-intensity life-events: 46–65; medium-intensity life-events: 66–97; high-intensity life-events: 98–135.

#### 2.2.3 PTSD check list-civilian version

The Chinese version of PCL-C was used to assess the PTSD symptoms of the respondents. The scale, compiled by Weathers, consists of 17 items divided into three categories: re-experience (5 items), avoidance (7 items) and hypervigilance (5 items) (40). Respondents were asked to rate PTSD symptoms based on “past stressful experiences” on a 1–5 Likert scale, with 1 = not at all, 2 = a little bit, 3 = moderately, 4 = quite a bit, and 5 = extremely. The total score of the scale ranges from 17 to 85, and the higher the total score, the higher the PTSD level. According to the literature, a score of 44 was used as the criterion for diagnosis of PTSD (41). This scale has shown good reliability and validity in Chinese adolescents (42). In the current study, the Cronbach’s α coefficient of the scale was 0.965.

#### 2.2.4 Patient health questionnaire depression scale

This scale is a self-rating scale for screening depressive symptoms based on the Diagnostic and Statistical Manual of Mental Disorders-IV (DSM-IV) and DSM-V, which has a good reference for the identification and diagnosis of depressive symptoms (43). The scale consists of 9 items, each of which is scored on a 4-point scale of 0–3, with a total score of 0–27. The higher the total score, the higher the depression level. According to literature reports, 10 points was used as the cut-off point to distinguish depression (44). The scale has been widely used in Chinese adolescents and has shown good reliability and validity (42). In this study, the Cronbach’s α of the scale was 0.91, indicating good internal consistency.

### 2.3 Quality control

First of all, the purpose, use and response requirements of the survey were explained in the questionnaire guidance. Respondents were required to answer carefully if they agreed to participate in this survey, and each option was not omitted to ensure the integrity and reliability of the questionnaire data collection. Secondly, the answering duration and response of each questionnaire provided by the questionnaire star were strictly screened. A total of 144 invalid questionnaires were excluded. The criterion for invalid questionnaires was that the answering duration was <5 min or there were a large number of missing answers or too many similar answers in the questionnaire.

### 2.4 Data analyses

SPSS 22.0 software (IBM SPSS Statistics, New York, United States) was used for descriptive analysis, correlation analysis, one-way analysis of variance, Logistic analysis and *T*-test and so on. Continuous variables were expressed as mean ± standard deviation, and inter-group comparison was performed by independent sample *t*-test. Categorical variables were expressed as percentages (%), and inter-group comparison was made by chi-square test. The Logistic regression model was used to analyze the influencing factors of depression and PTSD respectively, and to calculate the odd ratio (OR) among different subgroups. In the multivariate regression analysis, physical activity and life events were first included in the regression model as independent variables to assess their independent effects on depression and PTSD, respectively. Then, physical activity level (low level, medium level and high level) and life events (low intensity, average intensity, medium intensity and high intensity) were combined to form 12 combination variables (three groups of physical activity level × four groups of life events) to explore their combined effects on depression and PTSD. According to the literature (45), taking the group of high level of physical activity and very low intensity life events (ASLEC = 0–45) as references, the depression and PTSD were compared in groups with different levels of physical activity and different intensity of life events. Finally, a stratified analysis was conducted according to the levels of physical activity to assess the differences in the correlation between life events and depression and PTSD among different levels of physical activity. *p* < 0.05 was considered statistically significant.

## 3 Results

### 3.1 Comparison of demographic characteristics with detection rates of depression and PTSD

A total of 1,552 valid samples were included in this survey, ranging from 16 to 24 years old (19.37 ± 3.18), including 795 boys (51.22%), 171 ethnic minority students (11.02%) and 186 high school students (11.98%). In terms of the living habits of the respondents, 604 respondents (38.92%) slept <8 h per day, and 201 respondents (12.95%) smoke. In addition, 13.34, 38.92 and 47.74% of respondents achieved high, medium and low levels of physical activity, respectively. In terms of the family situation of the respondents, more than half of the respondents were from rural areas (52.06%), and 6.70% of respondents were from single-parent/ reorganized families. The proportion of those who considered their family economic status to be good, medium and poor was 22.55, 55.73 and 21.72%, respectively.

Chi-square test showed that women had higher detection rates of depression [*χ*^2^ (1) =22.35, *p* < 0.01] and PTSD [*χ*^2^ (1) =19.35, *p* < 0.01]. The detection rate of depression among ethnic minority students was higher than that of Han students [*χ*^2^ (1) = 9.35, *p* = 0.03] (Table 1). When the respondents slept for 8 h or more per day, the detection rate of depression was extremely significantly decreased [*χ*^2^ (1) = 16.09, *p* < 0.01], and the detection rate of PTSD was also significantly decreased [*χ*^2^ (1) = 8.14, *p* = 0.04]. In addition, the detection rate of depression in the groups of medium-level [*χ*^2^ (2) = 26.11, *p* < 0.01] and high-level [*χ*^2^ (2) = 24.92, *p* < 0.01] physical activity were relatively lower, and the detection rate of PTSD showed a similar trend. Depression [*χ*^2^ (1) = 7.97, *p* = 0.05] and PTSD [*χ*^2^ (1) = 8.97, *p* = 0.04] were lower in non-smokers. There were higher detection rates of depression [*χ*^2^ (1) = 30.91, *p* < 0.01] and PTSD [*χ*^2^ (1) = 41.01, *p* < 0.01] in students from single-parent/reorganized families. The depression detection rate of the students with good [*χ*^2^ (2) = 22.07, *p* < 0.01] and average family economic status [*χ*^2^ (2) = 18.99, *p* < 0.01] was lower than that of those with poor family economic status, and the PTSD detection rate also tended to be consistent.

**Table 1:** Comparison of demographic information, depression and PTSD among respondents.

Items	*n* (%)	Depression		PTSD	
		*n* (%)	*p*	*n* (%)	*p*
Gender					
Male	795(51.22)	165(20.75)		60(7.55)	
Female	757(48.78)	219(28.93)	<0.01	101(13.34)	<0.01
Age/years					
16–18 (excluding 18)	160(10.31)	40(25.00)		15(9.35)	
18–24 (including 18)	1,392(89.69)	344(24.71)		146(10.49)	
Nationality					
Han	1,381(88.98)	332(24.11)		139(10.07)	
Ethnic minority	171(11.02)	52(30.41)	0.03	22(12.87)	
Education level					
High school	186(11.98)	50(26.88)		23(12.36)	
College	1,366(88.02)	334(24.45)		138(10.10)	
Average daily sleep time					
< 8 h	604(38.92)	189(31.29)		81(13.41)	
≥ 8 h	948(61.08)	195(20.57)	<0.01	80(8.44)	0.04
Physical activity					
Low	741(47.74)	228(30.77)		105(14.17)	
Medium	604(38.92)	116(19.21)	<0.01	41(6.79)	<0.01
High	207(13.34)	40(19.61)	<0.01	15(7.25)	<0.01
Smoking					
Yes	201(12.95)	58(28.86)		28(14.93)	
No	1,351(87.05)	326(24.13)	0.05	133(9.84)	0.04
Family economic status					
Good	350(22.55)	71(20.29)	<0.01	24(6.86)	<0.01
Medium	865(55.73)	184(21.27)	<0.01	81(9.36)	<0.01
Poor	337(21.72)	129(38.27)		56(16.61)	
Single-parent/reorganized family					
Yes	104(6.70)	38(36.53)	<0.01	22(21.16)	<0.01
No	1,448(93.30)	346(23.89)		139(9.60)	
Home address					
City	744(47.94)	189(25.39)		81(10.89)	
Village	808(52.06)	195(24.13)		80(9.90)	
Total	1,552	384(24.74)		161(10.37)	

The ethnic minorities in this survey include Hui, Uyghur, Kazakh, Tujia, Mongolian, and so on.

### 3.2 Comparison of life events experiences

In terms of the total ASLEC score (Table 2), college students (*t* = 4.90, *p* = 0.03), smokers (*t* = 4.19, *p* = 0.04) and students from single-parent/reorganized families (*t* = 10.90, *p* < 0.01) experienced more life events. In addition, the total ASLEC scores of students with good, medium and poor family economic status also showed significant differences (*F* = 22.36, *p* < 0.01). After comparing the scores of all factors in the ASLEC, it was found that the scores of academic stress factor were significantly different among groups of high-level, medium-level and low-level physical activity (*F* = 15.30, *p* < 0.01). Interpersonal factors (*F* = 11.21, *p* < 0.01) and academic stress factors (*F* = 10.69, *p* < 0.01) also had significant differences among the students with three types of family economic status. In addition, Students from single-parent/reorganized family had higher scores in interpersonal factor (*t* = 9.89, *p* < 0.01), academic stress factor (*t* = 11.68, *p* < 0.01) and loss factor (*t* = 10.17, *p* < 0.01).

**Table 2:** Comparison of respondents’ life events experiences [Mean (SD)].

Items	Interpersonal	Learning stress	Punishment	Loss	Health adaptation	Other	Total
Gender							
Male	7.63 (3.22)	9.30 (4.03)	6.71 (2.29)	3.20 (1.25)	2.87 (1.07)	3.25 (1.49)	33.33 (10.82)
Female	8.11 (4.07)	9.51 (3.83)	6.03 (3.01)	3.51 (1.14)	2.41 (1.21)	3.58 (1.07)	34.59 (11.94)
Age/years							
16–18 (excluding 18)	6.87 (2.35)	9.79 (3.40)	7.01 (2.70)	3.77 (1.33)	2.59 (1.35)	3.81 (2.01)	33.87 (13.75)
18–24 (including 18)	8.09 (4.01)	9.05 (3.18)	6.57 (3.11)	2.99 (1.21)	2.71 (1.21)	3.07 (1.81)	35.29 (10.51)
Nationality							
Han	7.19 (4.22)	9.18 (3.51)	6.12 (2.61)	3.19 (1.29)	2.30 (1.50)	3.15 (1.23)	32.19 (10.22)
Ethnic minority	7.80 (5.24)	9.70 (4.09)	6.73 (3.22)	3.82 (5.24)	2.81 (1.22)	3.70 (1.40)	33.90 (13.04)
Education level							
High school	8.05 (4.54)	8.05 (3.54)	6.00 (4.11)	3.45 (1.51)	3.05 (1.43)	3.12 (1.04)	32.15 (14.77)
College	7.96 (3.83)	9.97 (3.80)	7.57 (3.84)	2.93 (2.03)	2.46 (1.23)	3.85 (1.72)	35.96 (13.81)^*^
Average daily sleep time							
≥ 8 h	7.90 (3.91)	9.08 (3.91)	6.91 (3.70)	2.90 (1.92)	2.61 (1.22)	3.76 (1.72)	31.07 (10.91)
< 8 h	8.08 (4.00)	8.78 (3.00)	6.17 (3.10)	3.68 (1.01)	3.00 (1.74)	3.01 (1.11)	33.68 (12.09)
Physical activity							
Low	8.21 (4.71)	9.71 (4.05)^**^	6.21 (2.70)	3.21 (1.41)	2.28 (1.44)	3.23 (1.27)	34.18 (12.01)
Medium	7.68 (3.32)	8.68 (3.41)	6.37 (3.32)	3.18 (1.62)	2.67 (1.30)	3.72 (1.33)	32.88 (11.39)
High	7.70 (3.64)	8.70 (3.25)	6.78 (3.13)	2.79 (1.74)	2.24 (1.23)	3.50 (1.61)	32.49 (10.14)
Smoking							
Yes	8.28 (3.90)	9.38 (3.90)	7.29 (3.10)	3.68 (1.91)	3.18 (1.20)	3.22 (1.81)	34.97 (13.90)^*^
No	7.67 (4.07)	8.79 (3.44)	6.66 (4.01)	3.23 (2.02)	2.70 (1.07)	3.69 (1.57)	32.07 (11.00)
Family economic status							
Good	7.06 (3.52)	8.26 (3.79)	6.02 (2.55)	3.16 (1.40)	2.41 (1.51)	3.06 (1.44)	31.00 (10.26)
Medium	7.75 (4.03)	8.65 (3.70)	6.74 (3.03)	3.31 (2.01)	2.15 (1.13)	3.77 (1.54)	33.71 (12.01)
Poor	**8.17 (3.73)** ^**^	**9.67 (3.55)** ^**^	6.99 (2.78)	3.88 (1.72)	2.60 (1.11)	3.97 (1.70)	39.17 (13.13)^**^
Single-parent/reorganized family							
Yes	8.44 (4.23)^**^	9.94 (4.12)^**^	6.98 (2.48)	4.14 (1.43)^**^	2.74 (1.19)	4.86 (1.21)	39.04 (12.21)^**^
No	7.18 (3.80)	8.18 (3.84)	6.19 (2.01)	3.10 (1.18)	2.18 (1.22)	2.78 (1.81)	30.21 (11.00)
Home address							
Village	8.14 (3.22)	9.19 (4.09)	6.83 (3.02)	3.19 (1.21)	2.17 (1.23)	3.18 (1.23)	34.14 (12.20)
City	7.88 (3.51)	9.40 (3.59)	6.11 (3.42)	3.80 (1.52)	2.86 (1.52)	3.88 (1.50)	32.89 (13.11)

Data in black indicates statistical significance. *p < 0.05, **p < 0.01.

### 3.3 Analysis of influencing factors of depression and PTSD

Multivariate logistic stepwise regression analysis was performed with anxiety and PTSD as dependent variables, respectively. The results in Table 3 showed that, when depression was used as dependent variable, gender, nationality, average daily sleep time, physical activity, smoking, family economic status, single-parent/reorganized family and life events experiences entered the equation, showing significant statistical significance. Further statistics showed that the risk of depression was 26% higher for women than for men, 17% higher for ethnic minorities than for Han, 32% higher for those who slept <8 h per day, 14% higher for smokers than non-smokers, 112% higher for single-parent/reorganized families than for non-single-parent/reorganized families, 51% higher for those with poor family economic status than for those with better economic status, and 27 and 131% higher for those who experienced medium- and high-intensity life events than for those who experienced low-intensity life events. In contrast, compared with those who engaged in low-level physical activity, those who regularly engaged in medium- and high-level physical activity had 49 and 53% lower rates of depression, respectively. When PTSD was taken as the dependent variable, gender, average daily sleep time, physical activity, smoking, family economic status, single-parent/reorganized family and life events experiences entered the equation, and the relationship between each variable and PTSD was similar to that of depression.

**Table 3:** Logistic regression analysis of influencing factors of depression and PTSD.

	Depression				PTSD			
Variables	*β*	SE	OR (95%CI)	*p*	*β*	SE	OR (95%CI)	*p*
Gender								
Male			1				1	
Female	0.27	0.09	1.26 (1.10, 1.69)	<0.01	0.21	0.08	1.20 (1.07, 1.55)	<0.01
Nationality								
Han			1					
Ethnic minority	0.22	0.06	1.17 (1.05, 1.67)	0.04				
Average daily sleep time								
≥ 8 h			1				1	
< 8 h	0.64	0.11	1.32 (1.09, 1.73)	<0.01	0.52	0.11	1.22 (0.97, 1.89)	<0.01
Physical activity								
Low			1				1	
Medium	−0.64	0.09	0.51 (0.42, 0.91)	<0.01	−0.16	0.14	0.45 (0.37, 0.81)	<0.01
High	−1.14	0.11	0.47 (0.32, 0.99)	<0.01	−0.84	0.13	0.41 (0.32, 0.95)	<0.01
Smoking								
Yes			1				1	
No	0.24	0.07	1.14 (0.72, 1.49)	0.03	0.34	0.07	1.15 (0.62, 1.36)	0.04
Family economic status								
Good			1				1	
Medium	0.09	0.06	0.93 (0.85, 1.01)	0.14	0.08	0.10	0.95 (0.88, 1.13)	0.18
Poor	1.07	0.12	1.51 (1.18, 3.22)	<0.01	1.21	0.12	1.40 (1.48, 3.02)	<0.01
Single-parent/reorganized family								
No			1				1	
Yes	0.94	0.05	2.12 (1.22, 3.54)	<0.01	1.21	0.21	1.84 (0.98, 2.13)	<0.01
Life events								
Low			1				1	
Average	0.11	0.11	1.11 (0.95, 1.21)	0.19	0.13	0.10	0.96 (0.91, 1.15)	0.12
Medium	0.28	0.12	1.27 (1.09, 2.91)	<0.01	0.24	0.14	1.17 (1.01, 2.21)	0.04
High	0.48	0.10	2.31 (1.36, 4.29)	<0.01	1.15	0.21	2.10 (1.36, 3.89)	<0.01

### 3.4 The combined effects of physical activity and life events on depression and PTSD

Different levels of physical activity (low, medium, high) and different intensity of life events (very low: 0–45; low: 46–65; medium: 66–97; high: 98–135) were combined to construct the joint variables, totaling 12 groups, with high-level physical activity × very low-intensity life events as the reference variable.

**Figure 1:**
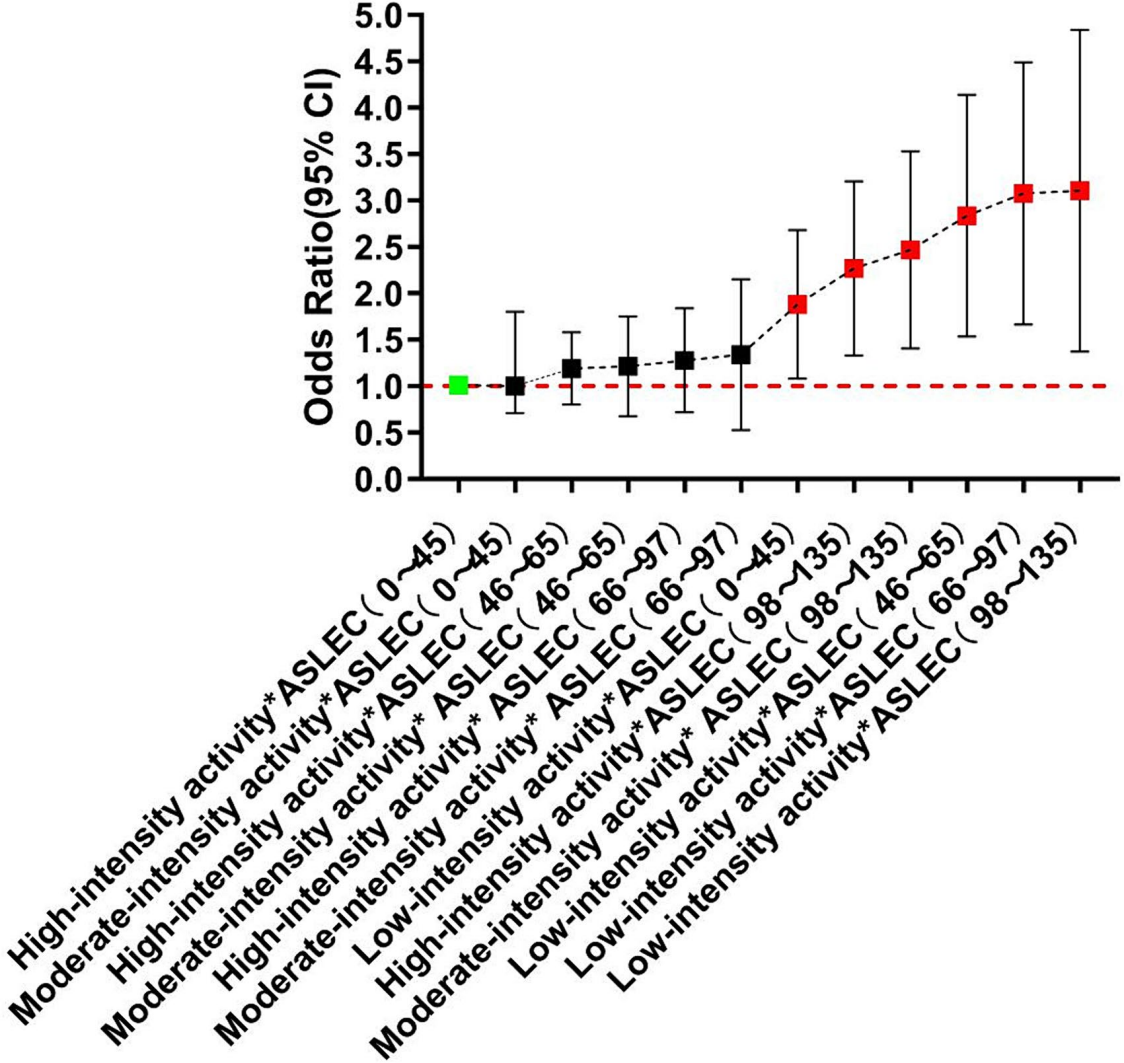
Combined effect of physical activity and life events on depression.

**Figure 2:**
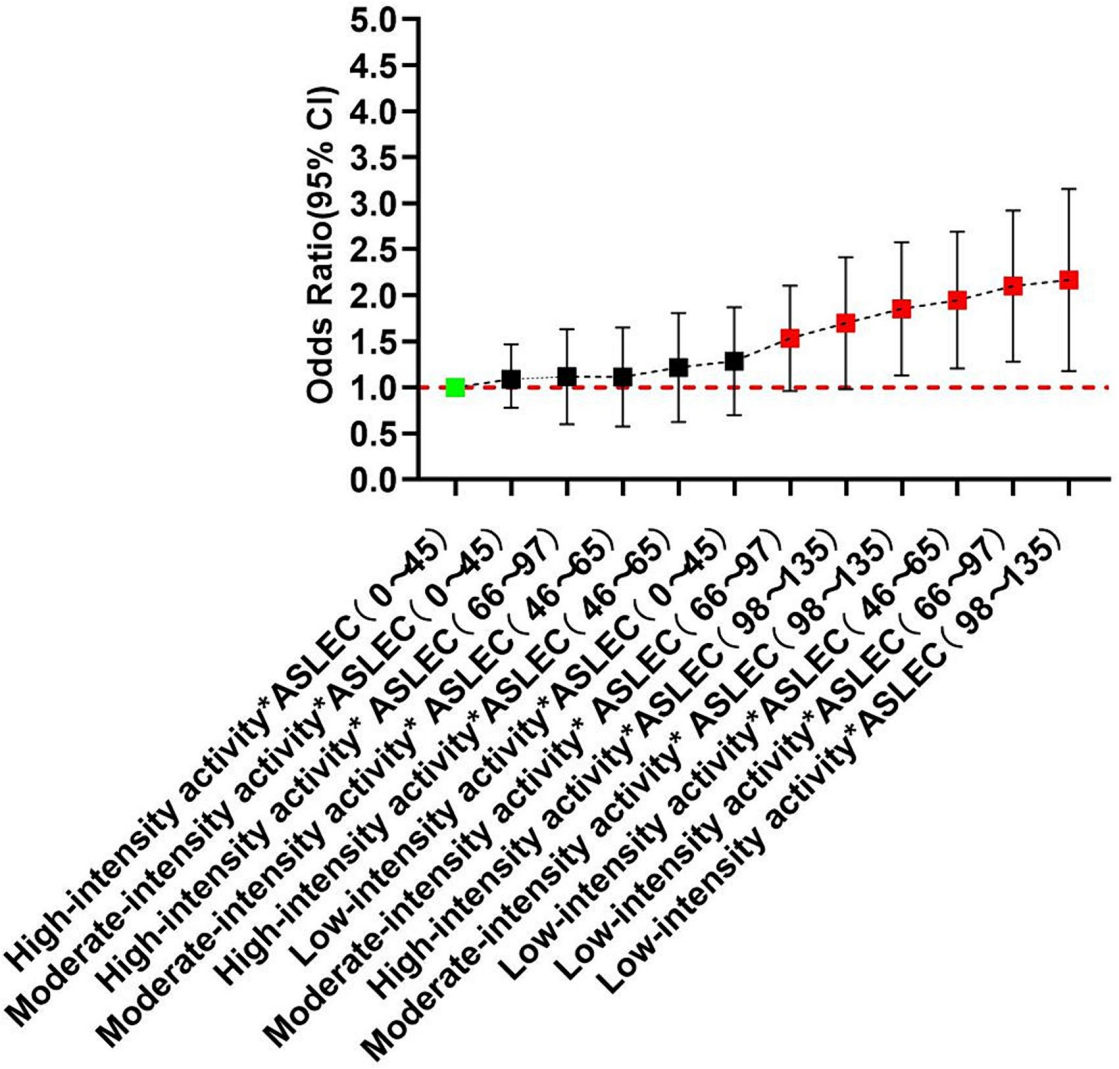
Combined effects of physical activity and life events on PTSD.

Sorted by the OR values of depression and PTSD detected in each group, the results showed that the risks of depression and PTSD increased with the increase of life events intensity and the decrease of physical activity level. Among them, the risks of depression and PTSD were the highest in students with low-level physical activity and high-intensity life events, which were 209 and 121% higher than the reference variable, respectively. Under the stimulation of high-intensity life events (ASLEC total score: 97–135), compared with low-intensity life events (ASLEC total score: 0–45), the risks of depression and PTSD increased by 117–209% and 65–121% in the students with high-, medium- and low-level physical activity, respectively (Figures 1, 2).

**Figure 3:**
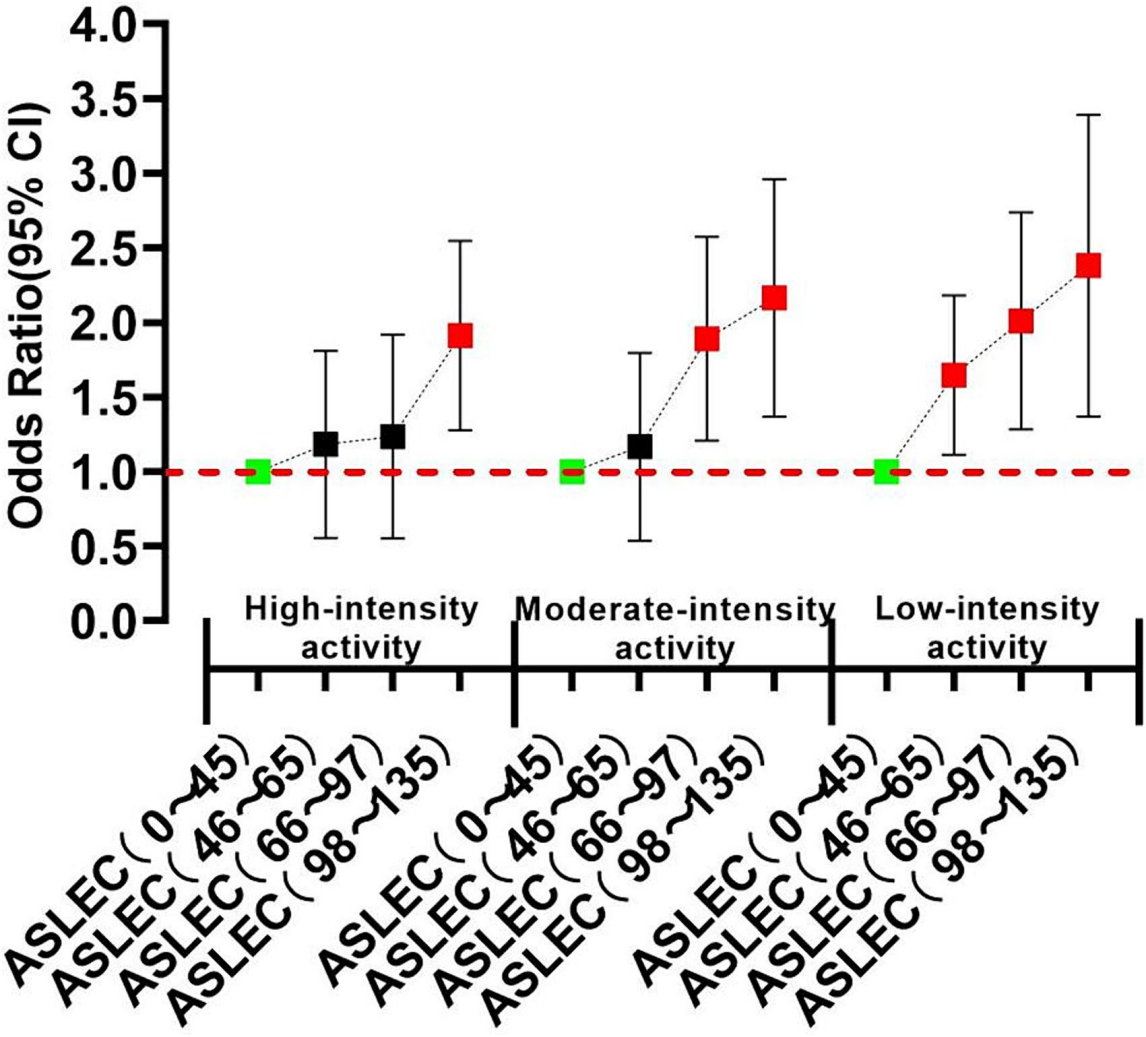
Stratified correlation analyses of physical activity and life events on depression.

**Figure 4:**
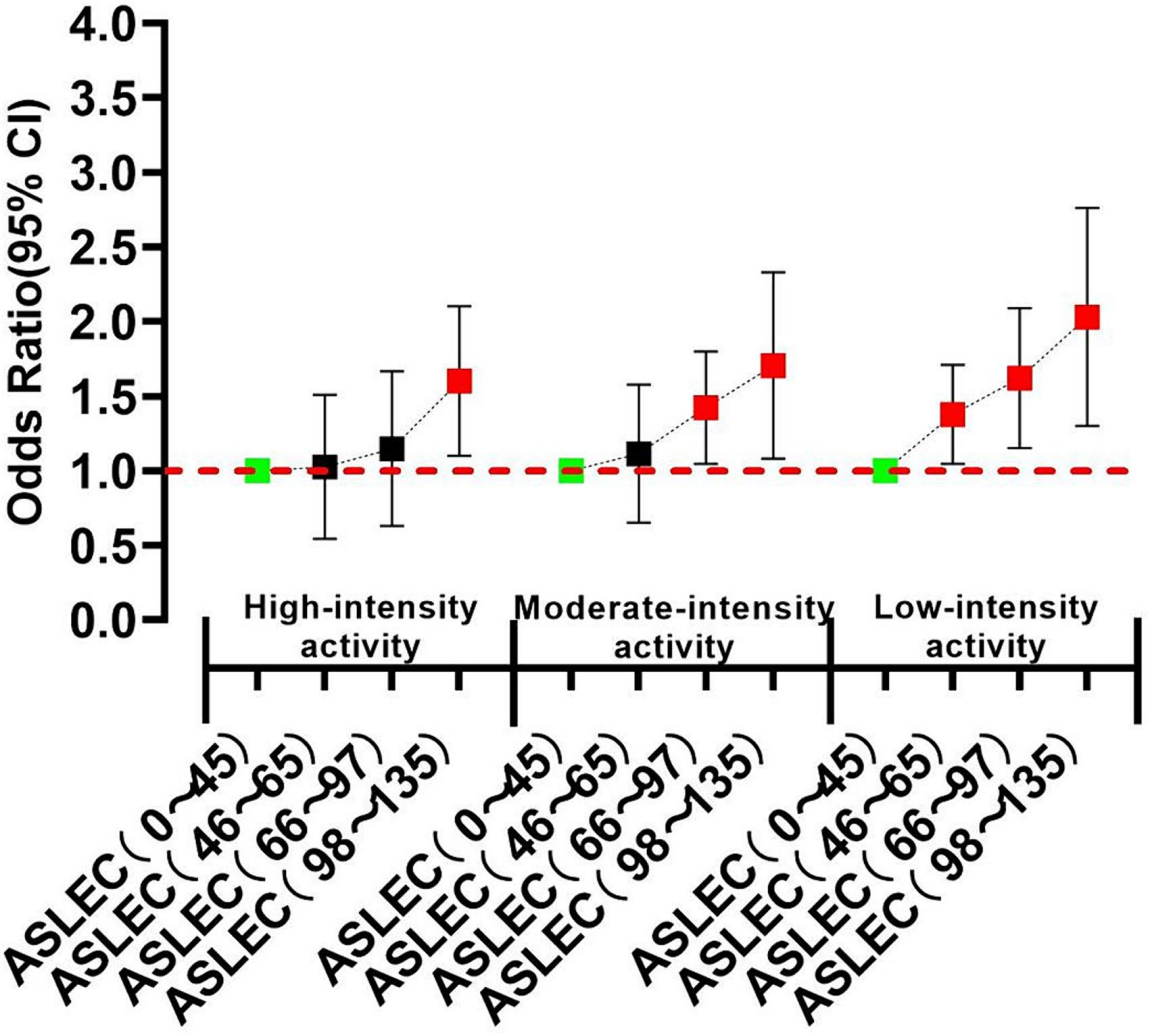
Stratified correlation analyses of physical activity and life events on PTSD.

Stratified analysis showed that, in different physical activity groups, life events experiences showed a similar trend to depression and PTSD, but the threshold of life events induced these psychological risks changed. In high-level physical activity group, only high-intensity life events (ASLEC total score: 98–135) were risk factors for depression and PTSD. However, in medium-level physical activity group, the threshold dropped to medium-level life events (ASLEC total score: 66–97). In low-level physical activity group, low-intensity life events (ASLEC total score: 46–65) could induce significant depression and PTSD (Figures 3, 4).

## 4 Discussion

### 4.1 Effects of life events on individual depression and PTSD

In this study, a cross-sectional survey of depression and PTSD prevalence among 1,552 Chinese students aged 16–24 years showed a prevalence of PTSD of 10.39%, which was basically consistent with the a meta-analysis of 11% lifetime PTSD among adolescents (46). The present study also found that 24.19% of the respondents met the criteria for depression. It was also found that the prevalence of depression in adolescents after trauma exposure was 24.2% (47) or the detection rate of depression was 17.0% in adolescent from Shanghai, China (48). The reason for the inconsistency might be related to the age difference of respondents. The median age of the respondents surveyed by Yu et al. (48) was only 13 years old, while the age of the respondents in this study was 19.37 ± 3.18. The present study also found gender differences in depression and PTSD detection rates, with higher detection rate in women compared to men. Depression and PTSD detection rates were also related to individual behaviors such as average daily sleep time, physical activity and smoking, as well as family economic status and family structure (single-parent/reorganized family), which were also confirmed by other studies (17, 39, 49). This study also found that life events exposure (ASLEC total score ≥ 66) was an important risk factor for depression and PTSD. Life events are not only associated with depression, anxiety, stress and PTSD symptoms (2–5, 11–13), but also important predictors of suicide (50) and dementia (51). Psychological findings explain the mechanism of life events affecting mental health from two aspects: on the one hand, life events indirectly affect mental health through some mediating variables; on the other hand, life events directly have a negative impact on mental health as stressors. For example, life events could not only induce depression through the mediating variable of attribution style (52), but also have an indirect effect on depression through the chain mediation of attribution-self-esteem (53). Loneliness played a mediating role in the relationship between negative life events and self-harm, suggesting that the influence of negative life events on self-harm might be caused by loneliness as a mediating variable (54). However, most studies have also confirmed that life events can directly induce depression (55, 56), anxiety (4, 57) and PTSD (3, 17) as stressors, and even directly predict suicide psychology (2, 50, 58).

Physiological findings provide more sufficient evidence for the mechanism of life events affecting mental health. It has been proven that anhedonia is a fundamental feature of depression, and life events stimulation can reduce the function of the brain’s reward system, and then aggravate the symptoms of anhedonia (59). Studies have found that major life events and stress stimulation can cause the dopaminergic reward system in the ventral tegmental (VTA) area of the midbrain to shut down automatically, resulting in anhedonia, despair, depression and other emotional experiences (60). Traumatic life events can impair functional connectivity between the midbrain periaqueductal gray matter and the amygdala, leading to PTSD symptoms (61). Stressful life events in childhood could lead to altered stress responses and abnormal activation of the HPA axis, making individuals develop psychopathology (6). Painful childhood experiences can also alter allele expression in serotonin transporter linked polymorphic region, thereby increasing the risk of depression in adulthood (62). In addition, experiencing too many negative life events in childhood can also change brain-derived neurotrophic factor (BDNF) gene polymorphisms, thus increasing the risk of depression in adulthood (63). In addition to directly inducing the expression of susceptible genes to cause depression, life events can also change gene expression without changing the DNA sequence through epigenetic means, thereby promoting the formation of depression (64–67).

Taken together, life events can directly or indirectly affect mental health through psychological and physiological mechanisms, and they are important risk factors for inducing depression and PTSD.

### 4.2 Combined effects of physical activity and life events on individual depression and PTSD

The present study found that the intensity of life events showed a dose–response relationship with depression and PTSD, and the change trend was the same in different physical activity levels, but the threshold of life events induced the two psychological risk gradually increased with the increase of physical activity level. That was, People who regularly engaged in high-level physical activity were at risk for depression and PTSD only after experiencing high-intensity life events, while those with low-level physical activity would have the risk of depression and PTSD only if they experience low-intensity life events. Combined correlation analysis further confirmed that higher level of physical activity could compensate to some extent for the negative effects of life events on individual depression and PTSD. This result can be explained by two aspects of psychological theory. One is the distraction hypothesis, which holds that individuals’ excessive focus on experienced life events can lead to unrealistic fantasies, stress, depression and PTSD symptoms such as flashbacks and re-experiences. Regular physical activity can distract people’s attention from adverse life events, vent negative emotions, relax mental stress, and then produce the effect of anti-anxiety and anti-depression (68). The other is self-efficacy theory, which holds that a person’s self-efficacy is positively correlated with his or her ability to control potential threats. Those who believe in their ability to manage or cope with potential threats (high self-efficacy) have less anxiety, depression and other negative psychology in the face of life events stimuli. Physical activity can improve self-efficacy (69, 70) and individual ability to adapt to internal and external stress, and then plays a role in resisting anxiety, depression and PTSD. The results of this study can also be explained by existing physiological studies. A large number of experimental results have shown that regular physical activity can regulate HPA axis activity, reduce the body’s responsiveness to stressors (71), increase nerve cell vitality and BDNF level (72), improve the function of reward system (increase the secretion of substances such as dopamine (DA), β endorphin and endocannabinoid (eCB) in midbrain) (73, 74), reduce the body’s inflammatory response (21, 75), improve brain oxygenation and energy supply (76), reduce oxidative stress damage caused by reactive oxygen species (77), and ultimately improve mood state, reduce adverse psychology such as depression, anxiety and PTSD induced by life events.

### 4.3 Limitations of the study

There are limitations in this study. First of all, this is a cross-sectional study, and causal inference cannot be made. The causal relationship between physical activity and depression and PTSD needs further research. Secondly, the respondents of this study mainly focused on Chinese students aged 16–24 years, so the interpretation of the study results needs to be cautious. Thirdly, although this study found that physical activity had a good regulatory effect on depression and PTSD induced by life events, it could not distinguish whether the regulatory effect was caused by physical activity before or after life events. Fourthly, the environment in which Chinese students aged 16–24 years live is very complex, including natural environment and social environment, and the factors that induce their depression and PTSD are also very extensive. However, the covariates controlled in this survey are very limited, resulting in some limitations in the study results. Nevertheless, the results of this study can still provide alternative methods and theoretical basis for mental health intervention of Chinese students aged 16–24 years and improving their quality of life.

## 5 Conclusion

A similar dose–response relationship was found for depression and PTSD symptoms in Chinese students aged 16–24 years as the intensity of life events stimulation increased. Physical activity can regulate this relationship, which is mainly reflected in the fact that the threshold stimulation intensity of depression and PTSD induced by life events increased with the increase of physical activity level, suggesting physical activity effectively buffered the adverse effects of life events stimulation on mental health.

## Data Availability

The raw data supporting the conclusions of this article will be made available by the authors, without undue reservation.
